# The podocyte-specific knockout of palladin in mice with a 129 genetic background affects podocyte morphology and the expression of palladin interacting proteins

**DOI:** 10.1371/journal.pone.0260878

**Published:** 2021-12-08

**Authors:** Nadine Artelt, Alina M. Ritter, Linda Leitermann, Felix Kliewe, Rabea Schlüter, Stefan Simm, Jens van den Brandt, Karlhans Endlich, Nicole Endlich

**Affiliations:** 1 Institute for Anatomy and Cell Biology, University Medicine Greifswald, Greifswald, Germany; 2 Imaging Center of the Department of Biology, University of Greifswald, Greifswald, Germany; 3 Institute of Bioinformatics, University Medicine Greifswald, Greifswald, Germany; 4 Central Core and Research Facility of Laboratory Animals (ZSFV), University Medicine Greifswald, Greifswald, Germany; University of Houston, UNITED STATES

## Abstract

Proper and size selective blood filtration in the kidney depends on an intact morphology of podocyte foot processes. Effacement of interdigitating podocyte foot processes in the glomeruli causes a leaky filtration barrier resulting in proteinuria followed by the development of chronic kidney diseases. Since the function of the filtration barrier is depending on a proper actin cytoskeleton, we studied the role of the important actin-binding protein palladin for podocyte morphology. Podocyte-specific palladin knockout mice on a C57BL/6 genetic background (PodoPalldBL/6-/-) were back crossed to a 129 genetic background (PodoPalld129-/-) which is known to be more sensitive to kidney damage. Then we analyzed the morphological changes of glomeruli and podocytes as well as the expression of the palladin-binding partners Pdlim2, Lasp-1, Amotl1, ezrin and VASP in 6 and 12 months old mice. PodoPalld129-/- mice in 6 and 12 months showed a marked dilatation of the glomerular tuft and a reduced expression of the mesangial marker protein integrin α8 compared to controls of the same age. Furthermore, ultrastructural analysis showed significantly more podocytes with morphological deviations like an enlarged sub-podocyte space and regions with close contact to parietal epithelial cells. Moreover, PodoPalld129-/- of both age showed a severe effacement of podocyte foot processes, a significantly reduced expression of pLasp-1 and Pdlim2, and significantly reduced mRNA expression of *Pdlim2* and *VASP*, three palladin-interacting proteins. Taken together, the results show that palladin is essential for proper podocyte morphology in mice with a 129 background.

## Introduction

More than 10% of the people worldwide are suffering from chronic kidney disease (CKD) and the tendency is still increasing [1]. In more than 75% of the diseased kidneys, a specific cell type in the filtration unit of the kidney, the podocyte, is damaged or lost [2].

Podocytes are highly specialized and post mitotic epithelial cells that cover the outer aspect of the glomerular capillaries and are part of the filtration unit of the kidney. For proper and size selective blood filtration, a complex interdigitation of the podocyte foot processes and a slit membrane in between is indispensable. Many publications in the past have impressively shown that such a 3D morphology of the podocyte is highly dependent on the actin cytoskeleton and their specific actin-binding proteins such as α-actinin-4 [3–7]. The loss or mutation of this actin-bundling protein, for example, leads to foot process effacement and severe proteinuria [5]. Furthermore, it was shown that proteins, involved in the nucleation as well as polymerization of actin filaments are essential for podocytes and therefore for kidney function.

In the present study, we have examined the role of the actin-binding and nucleating protein palladin which is important for the organization and stability of the actin cytoskeleton in many cell types [8–10]. An ubiquitous knockout (KO) of palladin in mice is lethal *in utero* around E15.5 and embryos develop cranial neural tube closure defects [10, 11]. Palladin is a scaffold protein consisting of immunoglobulin domains and proline-rich regions that interact with different proteins [8, 12] like the vasodilator-stimulated phosphoprotein (VASP) [13], the LIM and SH3 protein 1 (Lasp-1) [14] as well as the actin-binding protein α-actinin-1 [15]. VASP has been shown to be a regulator of actin assembly [13] and Lasp-1 regulates the cytoskeleton dynamics and cell migration [16, 17]. Furthermore, palladin interacts with ezrin which cross links cortical actin filaments to the plasma membrane [12, 18]. Another palladin binding protein, Pdlim2, is expressed in podocyte foot processes [19, 20]. It has been shown to interact with α-actinin-4 as well as angiomotin-like 1 (Amotl1) and seems to be involved in the pathogenesis of glomerular disease due to a regulation of the actin dynamics [20]. Beside this, different palladin isoforms exist which are expressed in a tissue- and development-dependent way [8, 14, 21]. However, the function of the specific isoforms is mainly unknown.

Our group has shown that palladin is specifically expressed in podocytes and plays an important role for podocyte morphology and dynamics *in vivo* as well as *in vitro* [22]. Furthermore, we recently found that cultured podocytes with reduced palladin expression developed only a few actin fibers and were more susceptible for a disruption of actin filaments after the treatment with the actin polymerization inhibitor cytochalasin D [23]. Additionally, the expression of the podocyte-specific and actin-binding proteins synaptopodin and α-actinin-4 were significantly reduced after the knockdown of palladin in podocytes [23].

Studying the podocyte-specific palladin knockout in C57BL/6 mice (PodoPalldBL/6-/-), we observed changes of the morphology of the glomeruli. These PodoPalldBL/6-/- mice developed glomeruli with dilated capillaries at 6 months of age as well as a mild broadening (effacement) of the podocyte foot processes [23]. Since the effect of a specific knockout is highly dependent on the genetic background of the mice and C57BL/6 mice are known to be robust against kidney damage to some extent, we wanted to study the effect of palladin in mice with a 129 genetic background (PodoPalld129-/- mice), which is known to be more sensitive against kidney damage in many studies [24–26]. Therefore, we backcrossed PodoPalldBL/6-/- mice with the 129 strain and studied the morphological changes of the glomeruli and podocytes in 6 and 12 months old mice. Moreover, we analyzed the expression of the palladin-binding partners Pdlim2, Lasp-1, Amotl1, ezrin as well as VASP.

## Materials and methods

### Generation of PodoPalld129-/- mice

PodoPalldBL/6-/- mice were generated as described previously [23]. These mice have a C57BL/6 background and were backcrossed to the 129 background using 129S2/SvPasCrl mice (Charles River Laboratories, Wilmington, MA, USA) and the *“speed congenics”* approach [27]. After 8x backcrossing, the new mouse line was established.

Podocyte-specific palladin knockout mice with 129 background (PodoPalld129-/-) and heterozygous Cre-recombinase expression were used for experiments. Mice without Cre-recombinase expression were used as controls (PodoPalld129+/+). Experiments were done with 6 and 12 months old male PodoPalld mice (at least n = 3 of each group). Genotyping of mice was performed with Phire^®^ Animal Tissue Direct PCR Kit (Finnzymes/Thermo Fisher Scientific) in accordance to the manufacturer’s instructions using primers shown in S1 Table.

All prerequisites of the German animal protection law were met and experiments were performed in accordance with the guidelines of the federal agencies in Mecklenburg-Western Pomerania (LALLF M-V). The responsible ethics committee within the LALLF M-V approved the experiments with mice. For kidney removal, mice were sacrificed by the use of barbiturate.

### Histology staining

The samples were dehydrated and embedded into paraffin by standard procedures. Paraffin sections (4 μm) were performed on a Leica SM 2000R (Leica Microsystems). After deparaffinization, sections were rehydrated and PAS and H&E stainings were performed by standard procedures. Sections were mounted in Eukitt (Fluka/Sigma-Aldrich, St. Louis, MO, USA) and imaged with an Olympus BX50 microscope (Olympus Europe, Hamburg, Germany).

### Immunofluorescence staining and immunohistochemistry of kidney sections

After deparaffinization, sections of mouse kidneys were rehydrated and unmasked in citrate buffer (0.1 M, pH 6.0) by heating for 5 min in a pressure cooker.

For immunofluorescence staining sections were blocked with blocking solution (2% FBS, 2% bovine serum fraction V, 0.2% fish gelatine in PBS) and incubated with the following primary antibodies overnight at 4°C: guinea pig anti-nephrin (GP-N2, ProgenBiotechnik GmbH, Heidelberg, Germany; 1:100), rabbit anti-podocin(P-037-2, Sigma-Aldrich Corporation, St. Louis (USA); 1:250), rabbit anti-ezrin(HPA021616, Sigma-Aldrich; 1:250), anti-p-Lasp-1 (kindly provided by Dr. Elke Butt, Würzburg; 1:500), rabbit anti-integrin-α-8 (sc-25713, Santa Cruz, Dallas, TX, USA; 1:100), rabbit anti-Pdlim-2, (sc-292831, Santa Cruz; 1:50). Bound antibodies were visualized with Cy3-conjugated secondary antibodies (Jackson ImmunoResearch Laboratories, West Grove, PA, USA; 1:600). Sections were embedded in Mowiol (Carl Roth, Karlsruhe, Germany). Images were acquired using a Leica TCS SP5 confocal laser scanning microscope (Leica Microsystems, Wetzlar, Germany).

For immunohistochemistry (IHC), the Vectastain kit (SP-2001, Vector Laboratories, Burlingame, CA, USA) was used following manufacturer’s instructions. Palladin was detected using rabbit anti-palladin (10853-1-AP, Proteintech Group, Manchester, UK; 1:850). Visualization was performed with DAB substrate kit (SK-4100; Vector Laboratories) followed by nuclear staining with hematoxylin and mounting in Eukitt (Sigma-Aldrich). In controls, PBS was used instead of primary antibody. Images were acquired using an Olympus BX50 microscope (Olympus Europe).

### Isolation of mouse glomeruli

Glomeruli were isolated with magnetic Dynabeads as described previously [28].

### RNA extraction and qRT-PCR analysis

Samples from glomeruli were processed in Tri-Reagent (Sigma-Aldrich) according to the manufacturer’s instructions. For cDNA synthesis, 1 μg of isolated total RNA was reverse transcribed using the QuantiTect Reverse Transcription Kit (Qiagen, Hilden, Germany). Quantitative real-time PCR (qRT-PCR) was performed on a LightCycler^®^ Nano (Roche Diagnostics GmbH, Mannheim, Germany) using iTaq Universal SYBR Green Supermix (Bio-Rad Laboratories GmbH, Hercules, CA, USA) and primers see S1 Table.

For qRT-PCR analysis we used the following number of mice per group: n = 5 of PodoPalld+/+ mice at 6 and 12 months of age, n = 6 and n = 7 of PodoPalld-/- mice at 6 and 12 months of age. Every control was compared to every PodoPalld-/- mouse for both age groups. The data were analyzed by standard methods [29] and the relative mRNA expression was calculated by normalizing values to the housekeeping gene *Rpl32*.

Asterisks indicate statistically significant differences (*p*<0.05) based on unpaired Student’s t-test or Mann-Whitney U test between the *Rpl32* and targets of interest using GraphPad Prism 8 (GraphPad, La Jolla, CA, USA). For the tests, n replicates (n≥5) were used and it was always checked for prerequisites such as normal distribution and similar variance between the measured groups.

### Glomerular morphology analysis of mouse kidney

For transmission electron microscopy, kidneys were embedded in EPON 812 (SERVA, Heidelberg, Germany). Ultrathin sections were cut and contrasted with 5% uranyl acetate and lead citrate. All grids were examined with a LIBRA^®^ 120 transmission electron microscope (Carl Zeiss Microscopy, Jena, Germany).

Scanning electron microscopy was performed according to Artelt *et al*. [30].

Furthermore, the presence of glomerular abnormalities was investigated more precisely using Richardson’s (Azur II/ Methylene blue) stained semithin sections of mouse kidneys. Glomeruli were categorized into (i) glomeruli with normal morphology, (ii) dilated capillaries and (iii) affected podocytes (podocytes with cyst and enlarged sub-podocyte space). The presence of dilated capillaries was verified by quantitative analysis of the capillary area per glomerulus on semithin sections.

We analyzed at least n = 3 mice of each group with a total of at least 30 glomeruli. Asterisks indicate statistically significant differences (*p*<0.05) based on two-way ANOVA analysis with FDR correction using GraphPad Prism 8.

## Results

### Confirmation of the podocyte-specific palladin knockout

To confirm that the backcrossed PodoPalld129-/- mice show a podocyte-specific knockout for palladin, we analyzed the animals by immunohistochemistry (IHC) and qRT-PCR. The IHC staining of PodoPalld129-/- mice revealed that the podocytes were palladin-negative at the age of 6 as well as of 12 months in contrast to the controls (S1A Fig). A faint signal for palladin (6 months: 0.26±0.14, 12 months: 0.19±0.05; mean±SD) was detected by qRT-PCR of isolated glomeruli which is due to a slight contamination with small vessels that still express palladin as shown in S1 Fig.

### PodoPalld129-/- mice show dilated capillaries as well as a reduced number of mesangial cells

**Fig 1:**
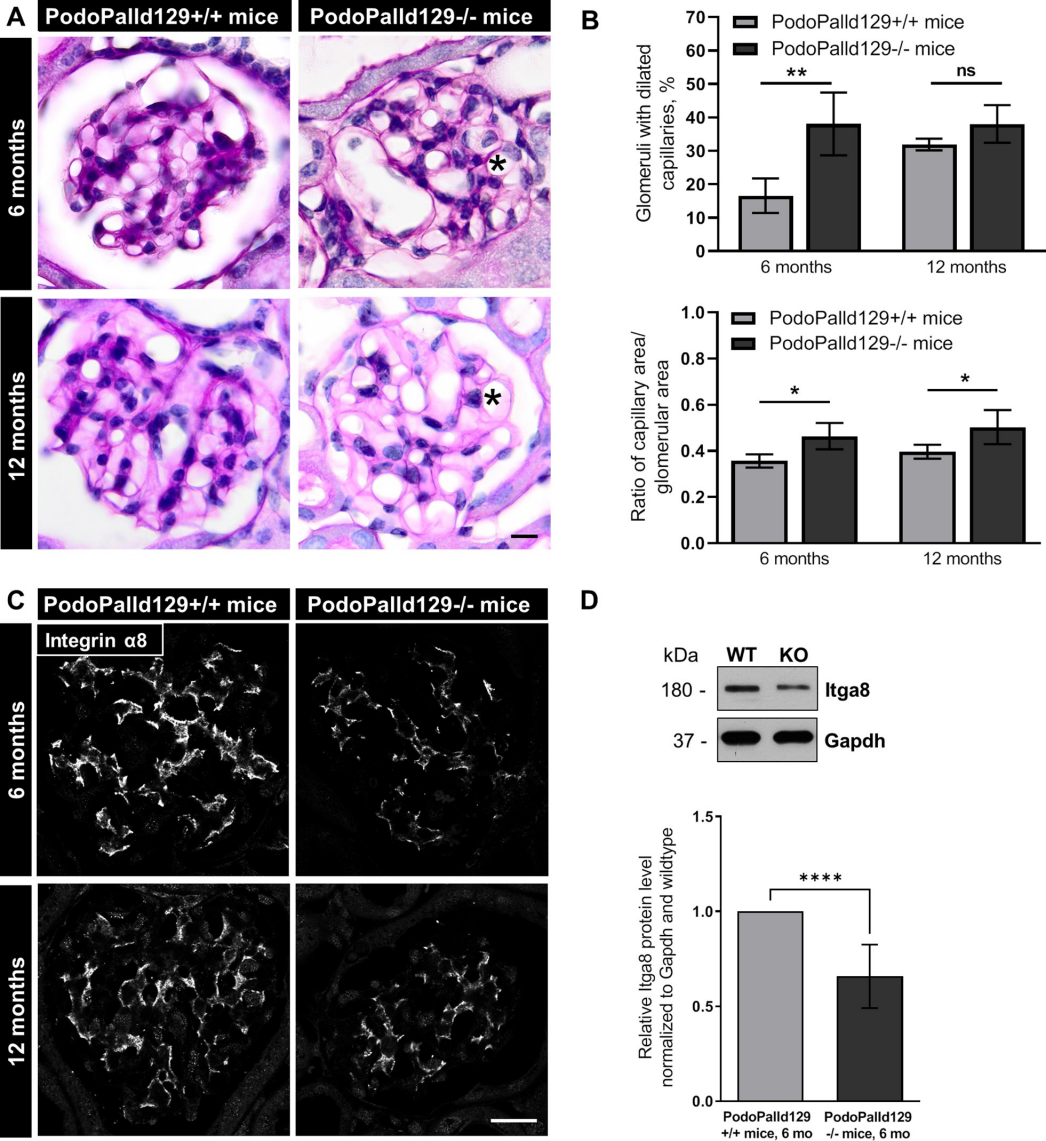
Glomeruli of PodoPalld129-/- mice have dilated capillaries and show a reduced expression of integrin α8. (A) The periodic acid staining of paraffin kidney sections revealed dilated capillaries in 6 and 12 months old PodoPalld129-/- mice (asterisks). Scale bar represents 10 μm. (B) Quantitative analysis confirmed that PodoPalld129-/- mice of both age groups have significantly more glomeruli with dilated capillaries than controls of the same age. (C) Immunofluorescence staining of kidney sections showed a marked reduction of integrin α8 expression in PodoPalld129-/- glomeruli. Scale bar represents 20 μm. (D) Western blot quantification of Itga8 showed a significant decrease in 6 months old PodoPalld129-/- glomeruli (KO) compared to the control (WT; 6 months old PodoPalld129+/+ glomeruli protein lysates). Data are presented as means ± SD; * *p*<0.05; ** p<0.01; **** p<0.001; ns, not significant; two-way ANOVA with FDR correction (B) or unpaired Student’s t-test (D).

We studied the glomerular morphology of two groups of mice, one group with an age of 6 months and the other with an age of 12 months by histological and ultrastructural analysis. All glomeruli of the PodoPalld129-/- mice developed severe dilatation of the capillaries as shown by a Periodic Acid Schiff (PAS) and Hematoxylin and Eosin (H&E) staining (Fig 1A and S2 Fig). We found that 38.1±9.4% (PodoPalld129-/-) vs. 16.5±5.2% (control; mean±SD, *p*<0.01) of the glomeruli at 6 month and 38.0±5.6% (PodoPalld129-/-) vs. 31.9±1.8% (control; mean±SD) at 12 months developed a dilatation of their capillaries (n = 3 animals and >60 analyzed glomeruli per group, Fig 1B). Quantitative analysis of the capillary area per glomerular area underlined that the capillaries of PodoPalld129-/- glomeruli were significantly enlarged (6 months: 0.46±0.06, *p*<0.05; 12 months: 0.50±0.07, *p*<0.05; mean±SD) compared to the control glomeruli (6 months: 0.36±0.02, 12 months: 0.40±0.03; mean±SD) (n≥3 animals and >30 glomeruli per group, Fig 1B). Since the dilatation of the glomerular tuft could be caused by a lower number of mesangial cells, we stained for the specific integrin α8 that is expressed in mesangial cells. Interestingly, we observed a marked reduction of the integrin α8 signal in 6 as well as in 12 months old PodoPalld129-/-mice compared to corresponding controls (Fig 1C) suggesting that palladin might influence the development or the survival of mesangial cells. The reduction of the Itga8 expression in PodoPalld129-/-mice was verified by Western blot (Fig 1D).

### Podocytes of PodoPalld129-/- mice develop morphological abnormalities

**Fig 2:**
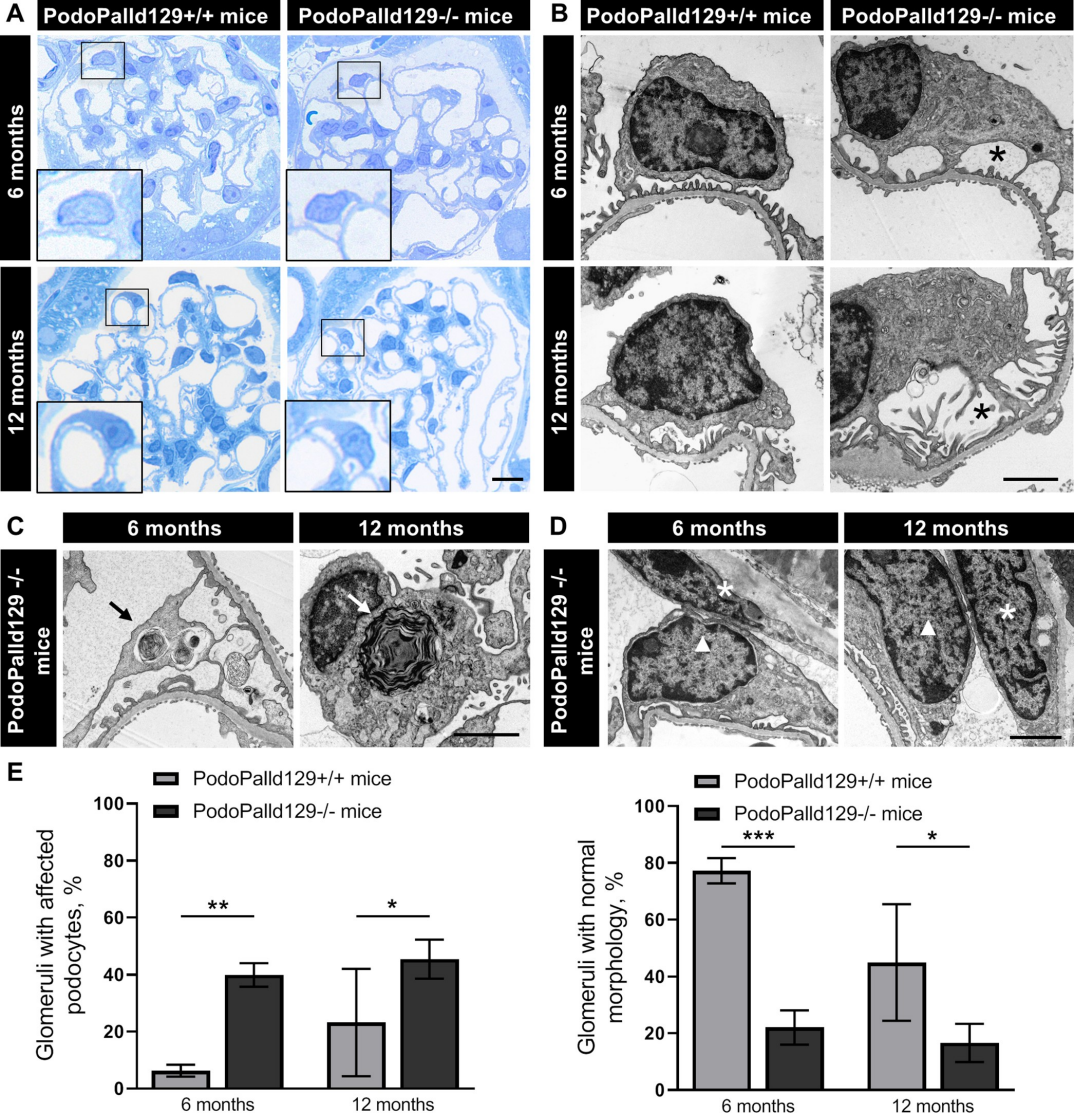
Podocytes of PodoPalld129-/- mice show morphological abnormalities. (A) In Richardson’s stained semithin sections of PodoPalld129-/- mice we have found podocytes with enlarged subpodocyte space compared with controls as illustrated in the higher magnifications. Scale bar represents 10 μm. (B) TEM images show podocytes with enlarged subpodocyte space in both age groups of PodoPalld129-/- mice (asterisks). (C, D) Podocytes with autophagosomes/laminar bodies (arrows) and contacts between parietal epithelial cells (asterisks) and podocytes (arrowhead) were found in TEM images of both age groups in PodPalld129-/- mice. Scale bars represent 2 μm. (E) PodoPalld129-/- mice had significantly more glomeruli with affected podocytes and correspondingly significantly fewer glomeruli without abnormalities than controls of the same age (mean±SD, * *p*<0.05, ** *p*<0.01 and *** *p*<0.001; two-way ANOVA with FDR correction).

To study the morphology of PodoPalld129-/- glomeruli in 6 and 12 months in more detail, we used Richardson’s stained semithin sections as well as ultrathin sections for the analysis by the transmission electron microscopy (TEM). Hereby, we found an enlargement of the so-called sub-podocyte space in animals with an age of 6 and 12 months, shown in Fig 2A and 2B. Furthermore, we identified several podocytes with autophagosomes and laminar bodies, respectively (Fig 2C). Moreover, we observed regions with a close contact of podocytes to parietal epithelial cells in both age groups of PodoPalld129-/- mice (Fig 2D). Quantitative analysis of PodoPalld129-/- glomeruli in comparison to the controls showed significantly more morphological alterations e.g. an enlarged sub-podocyte space, with an age of 6 months (39.9±4.1% vs. 6.3±2.1%; mean±SD, *p*<0.01) as well as of 12 months (45.4±6.8% vs. 23.2±18.9%; mean±SD, *p*<0.05 (n = 3 animals and >60 analyzed glomeruli per group, Fig 2E). Therefore, PodoPalld129-/- mice had correspondingly significantly fewer glomeruli with normal morphology than controls at 6 months (22.0±6.0% vs. 77.2±4.5%; mean±SD, *p*<0.05) as well as 12 months of age (16.6±6.7% vs. 44.9±20.6%; mean±SD, *p*<0.05) (n = 3 animals and >60 analyzed glomeruli per group, Fig 2E).

### The loss of palladin results in podocyte foot process effacement

**Fig 3:**
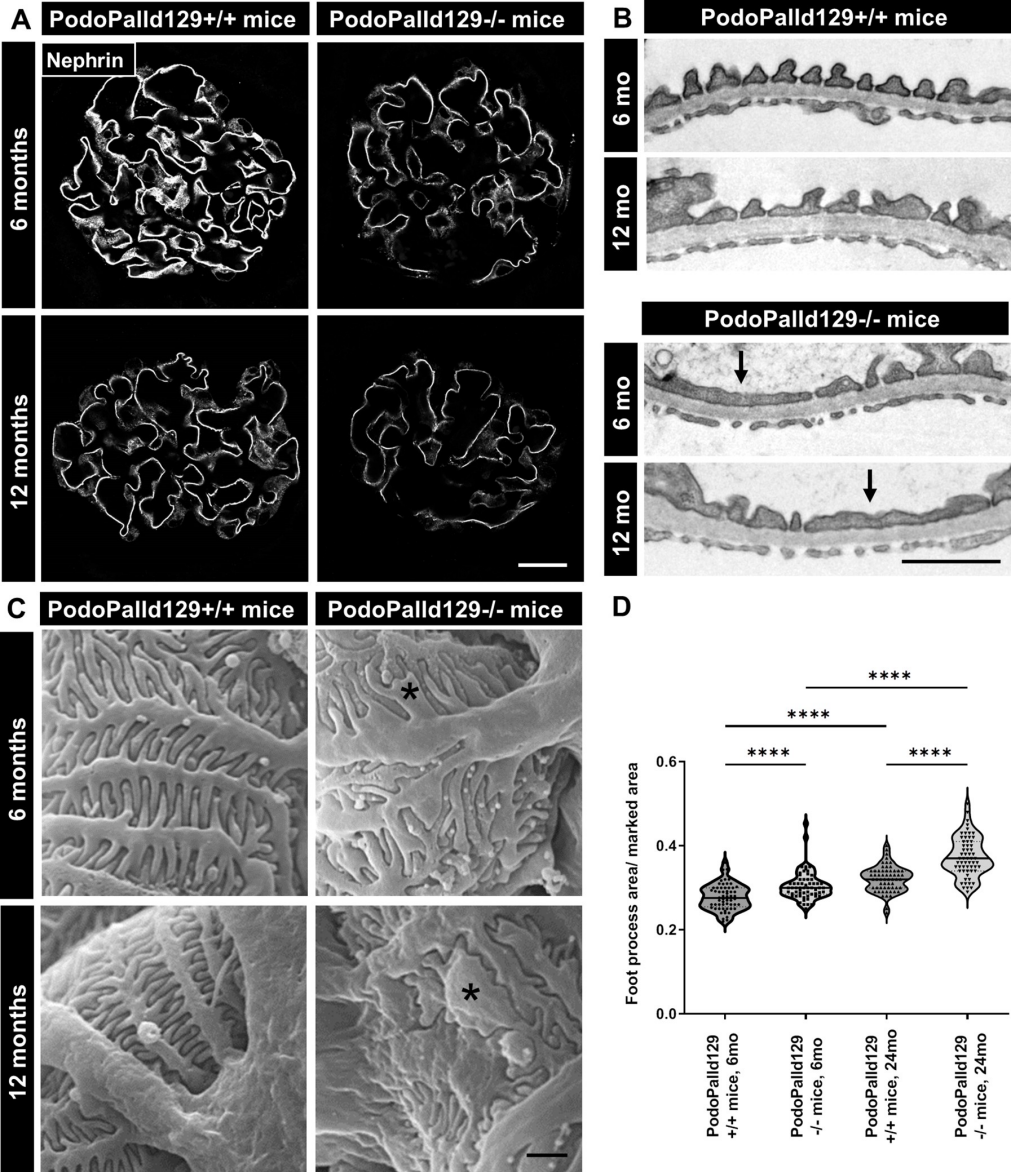
PodoPalld129-/- mice have effaced podocyte foot processes. (A) Immunofluorescence staining of kidney sections shows a reduced expression of the slit membrane protein nephrin in PodoPalld129-/- mice compared with corresponding controls. Scale bar represents 20 μm. (B) Transmission electron micrographs (arrows) and (C) scanning electron micrographs (asterisks) revealed strongly effaced podocyte foot processes in PodoPalld129-/- mice. Scale bars represents 1 μm. (D) Quantification of foot process area confirmed the increased podocyte foot process effacement in 6 months as wells as 12 months old PodoPalld129-/- mice compared with corresponding controls. Data are represented as violin plot (**** *p*<0.0001; two-way ANOVA with FDR correction).

Since PodoPalld129-/- mice developed morphological abnormalities we studied the expression of slit membrane proteins and foot process morphology in detail. Immunohistological staining showed that the slit membrane protein nephrin was significantly reduced in 6 as well as in 12 months old PodoPalld129-/-mice compared to the controls (Fig 3A), whereas expression of the nephrin mRNA was unchanged (6 months: 1.05±0.27, 12 months: 0.98±0.26; mean±SD) (S3 Fig). The same results were received for the slit membrane protein podocin (mRNA level 6 months: 1.09±0.32, 12 months: 1.07±0.37; mean±SD) (S3 Fig). A severe effacement of the podocyte foot processes was demonstrated by ultrastructural analysis using transmission electron microscopy (Fig 3B) and scanning electron microscopy (SEM, Fig 3C). Furthermore, quantification of the foot process area confirmed an increased podocyte foot process effacement in 6 months as wells as 12 months old PodoPalld129-/- mice compared to the corresponding controls (Fig 3D). Podocyte-specific loss of palladin does not result in proteinuria or albuminuria measured by dip stick and SDS electrophoresis (S5 Fig).

### Palladin knockout affects the expression of actin-binding proteins

**Fig 4:**
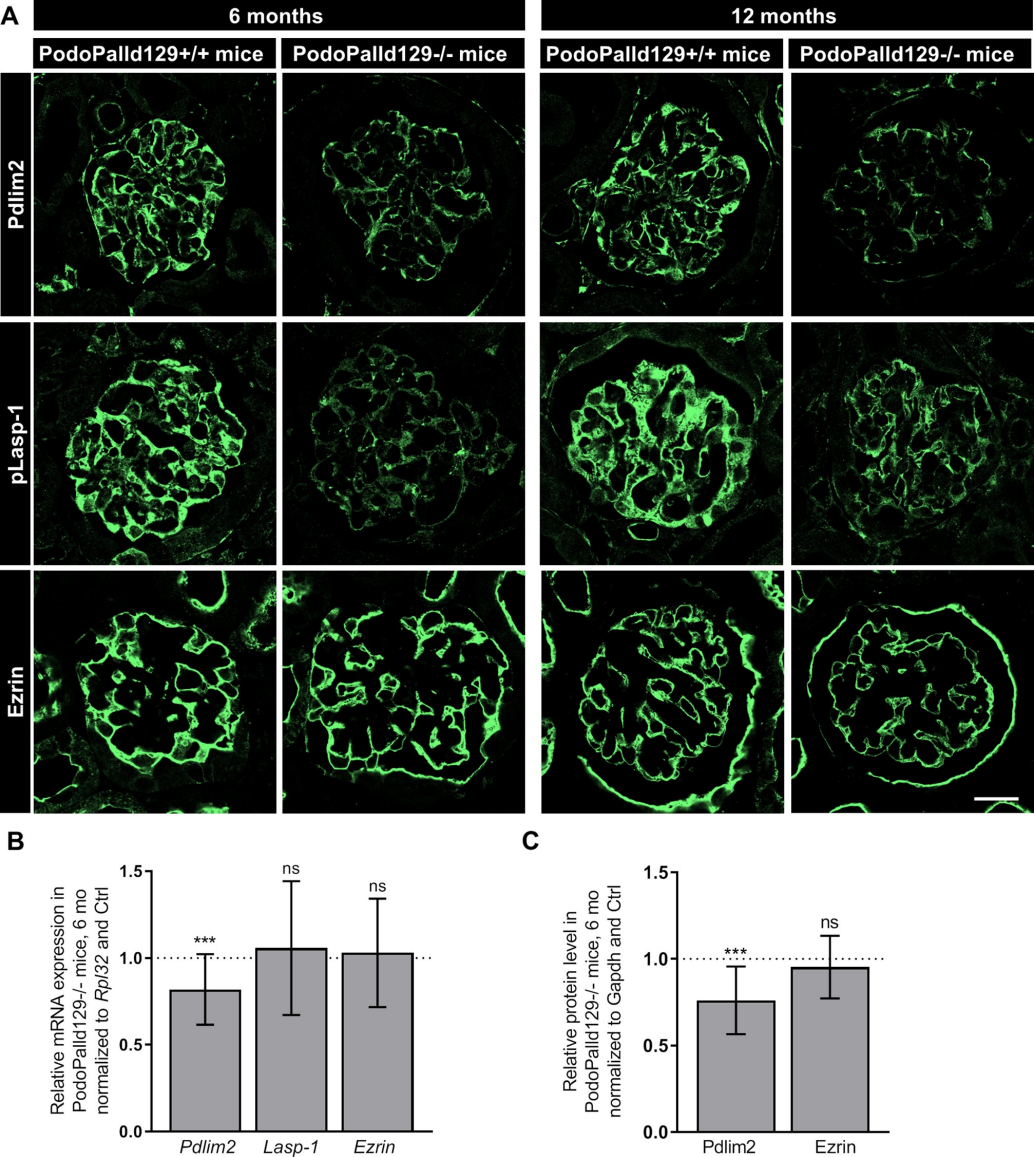
Analysis of palladin interacting proteins in PodoPalld129 mice. (A) Immunofluorescence stainings of kidney sections show a reduced expression of Pdlim2 and pLasp-1 in PodoPalld129-/- mice compared with corresponding controls. No difference was found for ezrin. Scale bar represents 20 μm. (B) A significant downregulation of *Pdlim2* mRNA in 6 months old PodoPalld129-/- glomeruli was found by qRT-PCR. *Lasp-1* and *Ezrin* were not significantly changed. (C) Relative protein level in 6 months old PodoPalld129-/- glomeruli normalized to Gapdh and protein lysates of PodoPalld129+/+ glomeruli. Data are presented as means±SD; *** p<0.001; ns, not significant; unpaired Student’s t-test.

Since podocyte morphology is highly dependent on the actin-cytoskeleton with their actin-binding proteins, we analyzed the role of palladin on the expression of essential actin- and palladin-binding proteins like Pdlim2, VASP, Lasp-1, ezrin and Amotl1, which were all expressed in podocytes. After the immunofluorescence staining of kidney sections, we found that Pdlim2 and phosphorylated Lasp-1 (pLasp-1) was markedly reduced in PodoPalld129-/- mice at the age of 6 as well as of 12 months compared with the controls (Fig 4A). In contrast, the protein expression of ezrin was unchanged (Fig 4A). Different antibodies against Lasp-1 and VASP showed unfortunately no reactivity on mouse tissue. mRNA expression analysis showed that *Pdlim2* was also significantly reduced in the glomeruli (6 months: 0.82±0.20, *p<*0.001; mean±SD), whereas *Ezrin* was unchanged (6 months: 1.03±0.31; mean±SD) (Fig 4B). This was also confirmed by Western blot quantification (Fig 4C). qRT-PCR analysis further showed a significant down-regulation of *VASP* mRNA level (6 months: 0.71±0.41, *p<*0.01; 12 months: 0.78±0.28; *p<*0.001; mean±SD) and an upregulation of *Amotl1* mRNA level (6 months: 1.51±0.47, *p<*0.001; 12 months: 1.32±0.57, *p<*0.01; mean±SD) compared to controls with the same age (S4 Fig).

## Discussion

Palladin, an essential actin-binding and regulating protein, is ubiquitously expressed in mammals [8, 12]. Recently, our group has shown that palladin is specifically expressed in podocytes, a post mitotic cell type in the kidney [22] where it plays an important role in cross-linking and stabilization of actin filaments [8, 31]. Additionally, we demonstrated that palladin is essential for a proper 3D morphology of podocytes as well as for the glomerular tuft formation *in vivo*, especially after the challenge of the mice with nephrotoxic serum (NTS), which is a well-established kidney disease model [23, 32].

It is well known that the genetic background of the mouse strain has an important influence on the severeness and development of kidney disease. As it was nicely shown by Steppan and colleagues, mouse strains have distinct vascular properties [33]. They have found that the blood pressure seems to be similar in 129S and C57BL/6 strains, however there are significant differences in vascular properties which might influence the severeness and onset of kidney diseases. Therefore, it is important to study the role of proteins in different and good characterized mouse strains.

Here we studied the influence of palladin not only in the mainly used C57BL/6 mice strain, which is often described to be robust against kidney damage, but also in the often more sensitive 129 genetic mouse strain [24–26]. For this purpose, we backcrossed the palladin knockout mice to the 129 background and analyzed the animals at an age of 6 months and 12 months. We have found that PodoPalld129-/- mice were more affected by the palladin knockout compared with PodoPalldBL/6-/-mice. In 6 month old knockout mice with a C57BL/6 background, approximately 20% of glomeruli showed dilated capillaries [23]. In contrast, glomeruli of 6 months old PodoPalld129-/-mice showed a significant increased dilatation of the glomerular tuft (38±9.4%) compared to the control mice (16.5±5.2%; mean±SD, *p*<0.01) at the same age. Interestingly, the number of glomeruli with dilated capillaries did not increase further in 12 months old PodoPalld129-/- mice, indicating that the glomerular phenotype developed already during the first 6 months. This analysis underlines the importance of the genetic background of animals with a specific knockout because it severely influences the phenotype.

To identify the reason for such a dilatation in PodoPalld129-/- mice, we investigated the presence of mesangial cells. Mesangial cells are contractile cells and play a key role in capillary loop formation [34]. Since there is an interaction and cross-talk between mesangial cells, endothelial cells and podocytes [34], we examined whether the palladin knockout in podocytes also has an indirect effect on mesangial cells. To investigate this we stained kidney sections with an antibody specific for mesangial cells against integrinα8 [35] and found a significant downregulation of the protein. This could also be observed by loss of mesangial cells in early stages of glomerulonephritis/-sclerosis when mesangiolysis and capillary expansion occurred [36, 37]. Since we found only a faint staining for this specific integrin in the glomeruli of PodoPalld129-/- mice in contrast to the control mice at the same age, we hypothesized that here the number of mesangial cells is reduced. Whether this is due to a developmental failure or caused by the loss of mesangial cells after birth is still unknown.

Beside a dilatation of the glomerular tuft, we found that the majority of PodoPalld129-/- podocytes developed an enlarged sub-podocyte space as well as cysts compared to the controls of the same strain and to podocytes of the PodoPalldBL/6-/- mice [23]. Ultrastructural analysis of the foot processes by scanning and transmission electron microscopy revealed that nearby 50% of the analyzed PodoPalld129-/- podocytes in mice with an age of 12 months developed such a severe phenotype. Moreover, this phenotype was already described by Kriz and colleagues in a variety of models [38, 39]. Based on descriptions in different podocyte-related diseases [40–42], our results lead to two assumptions. First, this phenotype could be caused by an imbalanced growth of the glomerular tuft due to a low number of mesangial cells and/or by podocyte hypertrophy to compensate lost podocytes which might be characteristic for this specific strain. Second, it could be that difference is vascular stiffness observed by Steppan and colleagues [33] leads to a higher risk of developing glomerular hypertension, especially already at a young age.

Furthermore, PodoPalld129-/- mice at 12 months of age showed more effaced foot processes compared to 6 months old mice. This finding is in nice agreement with the quantification of podocyte foot process morphology by super resolution microscopy as already described [30]. Here we could show that 6 and 12 months old PodoPalld129-/- mice possess a significant reduction of the filtration slit density (FSD) resulting insignificantly more effaced podocyte foot processes compared to the littermates [30]. In addition, in this study we could show that protein expressions of the slit diaphragm proteins nephrin and podocin were significantly downregulated in PodoPalld129-/- mice compared to corresponding controls.

Beside the morphological findings, we observed autophagosomes in PodoPalld129-/- podocytes which is a hallmark of autophagy, a self-repair mechanism of post mitotic cells [43]. Interestingly, patients suffering from IgA nephropathy and membranous nephropathy, both podocyte-related kidney diseases, showed also an increase of autophagosomes in podocytes [44, 45].

Moreover, ultrastructural analysis revealed an increased number of contacts between podocytes and parietal epithelial cells (PECs) in PodoPalld129-/- glomeruli. This is of specific interest since it is known that contacts between podocytes and PECs trigger the formation of tuft adhesions that are first committed lesions for focal segmental glomerulosclerosis. [46–48].

The podocyte foot process morphology is highly dependent on an intact actin cytoskeleton. Therefore, we studied the influence of palladin on the actin cytoskeleton in these specific knockout mice. Since it is described that palladin has specific binding-sites for proteins which are involved in actin dynamics and stability like Lasp-1 [14], Pdlim2 [19], ezrin [12], and VASP [13], we investigated the effect of the palladin knockout on the expression of these proteins. Although palladin is known to recruit Lasp-1to actin stress fibres and that the palladin knockdown in HeLa cells resulted in a reduction of Lasp-1 [14, 49], we have found that the *Lasp-1* mRNA expression was unchanged in PodoPalld129-/- podocytes. However, the phosphorylation of Lasp-1 was significantly reduced. This might influence the stability of the podocyte foot processes, since it was already shown that phosphorylation of Lasp-1 reduces the binding to F-actin *in vitro* [16, 50].

Furthermore, we have detected a significant down-regulation of Pdlim2 in podocytes of PodoPalld129-/- mice. Pdlim2 was already shown to be essential for the stability of the actin cytoskeleton in cultured podocytes as well as was regulated in patients suffering from glomerulopathies [20]. Interestingly, the Pdlim2-interacting partner Amotl1 [20], a protein which regulates the actin dynamic [51], was significantly up-regulated in PodoPalld129-/-glomeruli. Probably, Amotl1 is able to compensate the reduced Pdlim2 expression.

Another important actin-binding protein, VASP, which also regulates actin dynamics, was significantly down-regulated in 6 and 12 months old PodoPalld129-/- mice, indicating that palladin directly influence this protein via the binding site. In contrast, we observed no difference in the expression of ezrin, an actin-binding and plasma membrane cross-linking protein [52], between control and PodoPalld129-/- mice.

Taken together, this study demonstrates that palladin has an impact on the expression of the interacting proteins Pdlim2, pLasp and VASP and is important for capillary tuft formation and podocyte morphology.

## Supporting information

S1 Fig Generation of PodoPalld129-/- mice and confirmation of the podocyte-specific KO of palladin. (A) The specific palladin KO was confirmed by immunohistochemistry staining of paraffin kidney sections. PodoPalld129+/+ mice exhibit strong palladin-expressing podocytes (arrow). In contrast, there is no palladin signal (arrowhead) in PodoPalld129-/- podocytes. Scale bar represents 10 μm. (B) In addition, the palladin KO was verified by qRT-PCR (mean±SD, ***p<0.001; 6 months: Mann-Whitney U test, 12 months: unpaired Student’s t-test).
(TIF)

S2 Fig Glomeruli of PodoPalld129-/- mice have dilated capillaries. The hematoxylin and eosin staining of paraffin kidney sections showed dilated capillaries in 6 and 12 months old PodoPalld129-/- mice (asterisks). Scale bar represents 10 μm.
(TIF)

S3 Fig Analysis of slit membrane proteins of PodoPalld129 mouse glomeruli. (A) Quantitative analysis of nephrin mRNA levels in isolated glomeruli showed no significant difference between PodoPalld129-/- mice and controls (mean±SD; unpaired Student’s t-test). (B) Immunofluorescence staining of kidney sections reveal a slightly decreased expression of the slit membrane protein podocin in PodoPalld129-/- mice compared with corresponding controls. Scale bar represents 20 μm. (C) However, we found no significant difference of podocin mRNA in isolated glomeruli of PodoPalld129-/- and PodoPalld129+/+ mice (mean±SD; 6 months: Mann-Whitney U test, 12 months: unpaired Student’s t-test).
(TIF)

S4 Fig Quantitative analysis of mRNA level of palladin-interacting proteins. A significant downregulation of *Vasp* mRNA and upregulation of *Amotl1* mRNA in PodoPalld129-/- glomeruli was found by qRT-PCR. Data are presented as means ± SD; ** p<0.01; *** p<0.001; unpaired Student’s t-test.
(TIF)

S5 Fig Quantitative analysis of urine of PodoPalld129+/+ and PodoPalld129-/- mice. Equal volume of sterile urine from 6- and 12-months old PodoPalld129+/+ and PodoPalld129-/- mice were separated by SDS-Page and were subsequently stained using CBB (Coomassie Brilliant Blue). BSA was used as a charge control (lane 2). No albumin band is seen (dotted outline), in 6 months old PodoPalld129-/- (lane 4–6) as well as in 12 months old PodoPalld129-/- (lane 8–10) indicating no increased proteinuria in the PodoPalld129-/- mice. The major urinary proteins (MUPs) showed a strong signal between 15 kDa and 20 kDa.
(TIF)

S1 Table Primer for RT-PCR and qRT-PCR. (DOCX)
