# Supplementary figures and images for: The podocyte-specific knockout of palladin in mice with a 129 genetic background affects podocyte morphology and the expression of palladin interacting proteins
Source: PLoS One. 2021 Dec 8;16(12):e0260878. doi: 10.1371/journal.pone.0260878 (PMC8654177; doi:10.1371/journal.pone.0260878)

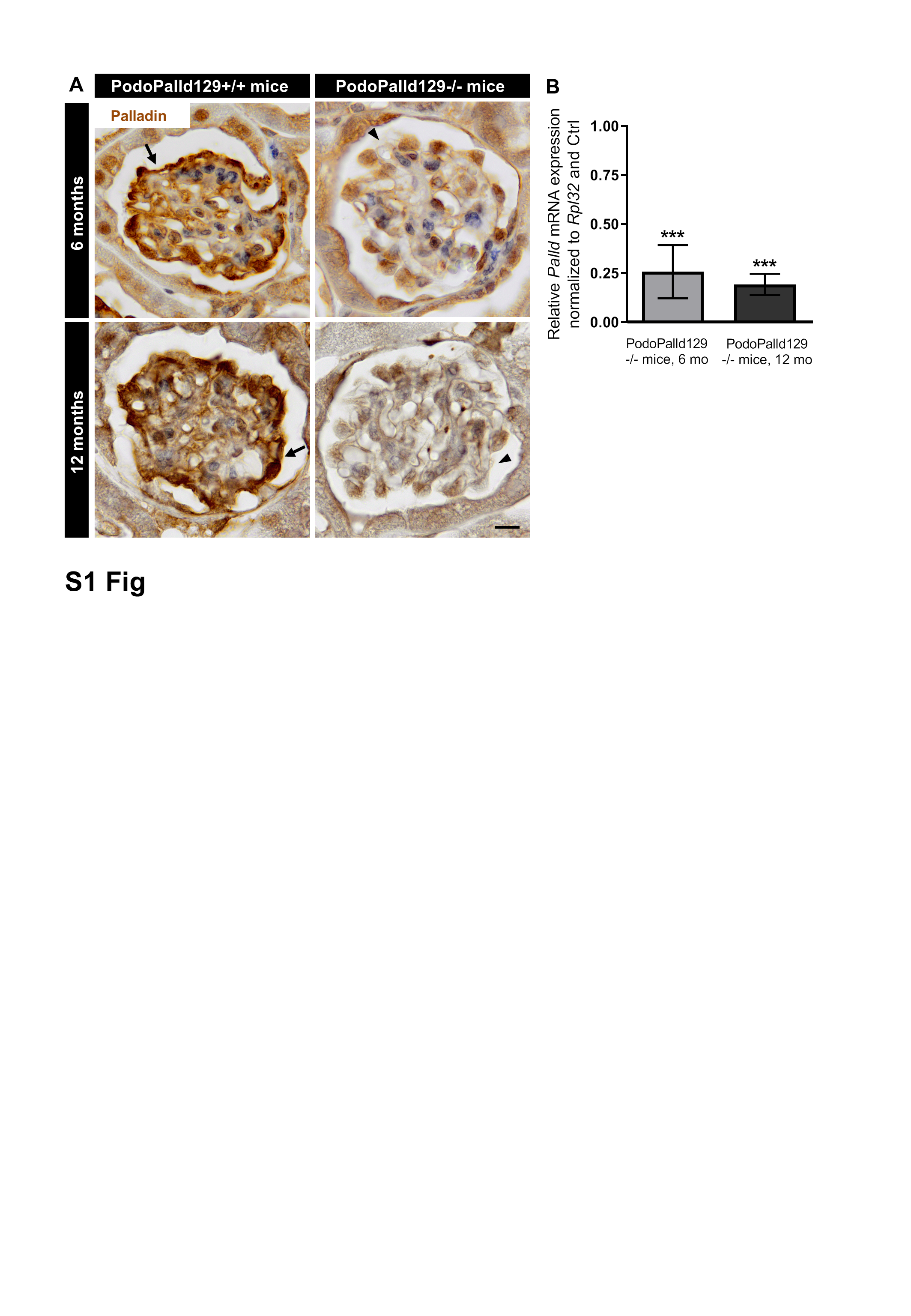

Supplement: S1 Fig — (A) The specific palladin KO was confirmed by immunohistochemistry staining of paraffin kidney sections. PodoPalld129+/+ mice exhibit strong palladin-expressing podocytes (arrow). In contrast, there is no palladin signal (arrowhead) in PodoPalld129-/- podocytes. Scale bar represents 10 μm. (B) In addition, the palladin KO was verified by qRT-PCR (mean±SD, ***p<0.001; 6 months: Mann-Whitney U test, 12 months: unpaired Student’s t-test). (TIF) [file pone.0260878.s001.tif]

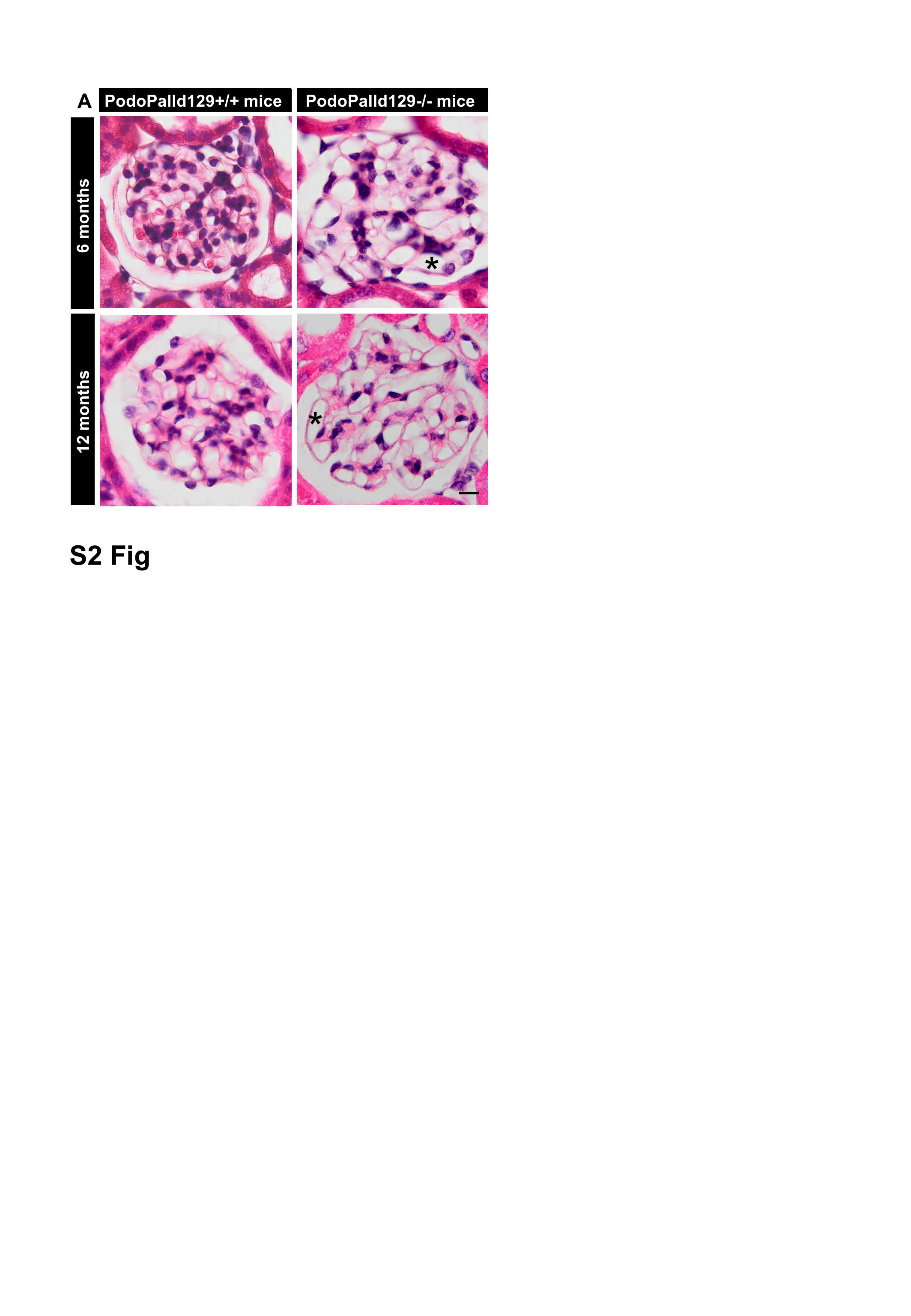

Supplement: S2 Fig — The hematoxylin and eosin staining of paraffin kidney sections showed dilated capillaries in 6 and 12 months old PodoPalld129-/- mice (asterisks). Scale bar represents 10 μm. (TIF) [file pone.0260878.s002.tif]

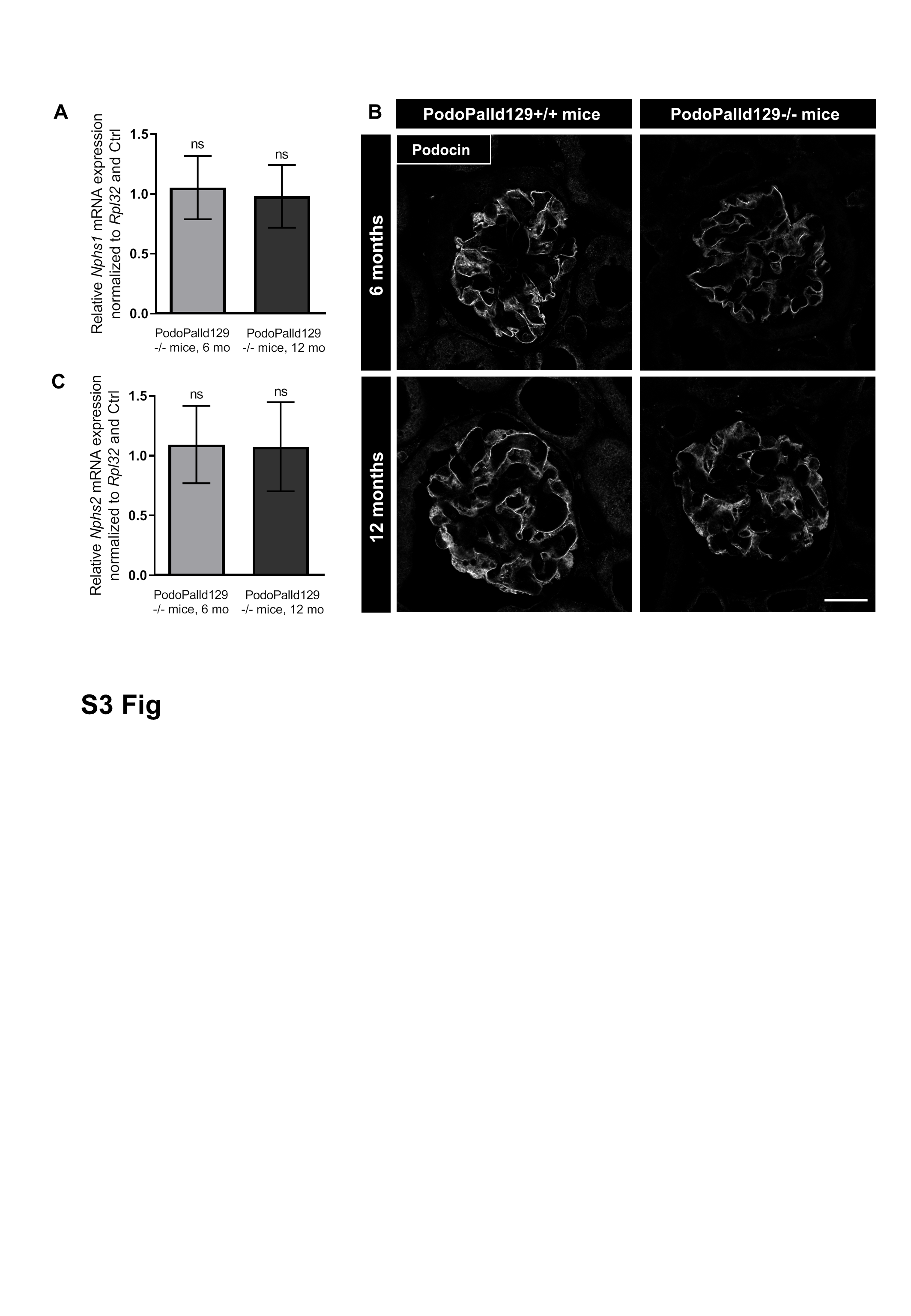

Supplement: S3 Fig — (A) Quantitative analysis of nephrin mRNA levels in isolated glomeruli showed no significant difference between PodoPalld129-/- mice and controls (mean±SD; unpaired Student’s t-test). (B) Immunofluorescence staining of kidney sections reveal a slightly decreased expression of the slit membrane protein podocin in PodoPalld129-/- mice compared with corresponding controls. Scale bar represents 20 μm. (C) However, we found no significant difference of podocin mRNA in isolated glomeruli of PodoPalld129-/- and PodoPalld129+/+ mice (mean±SD; 6 months: Mann-Whitney U test, 12 months: unpaired Student’s t-test). (TIF) [file pone.0260878.s003.tif]

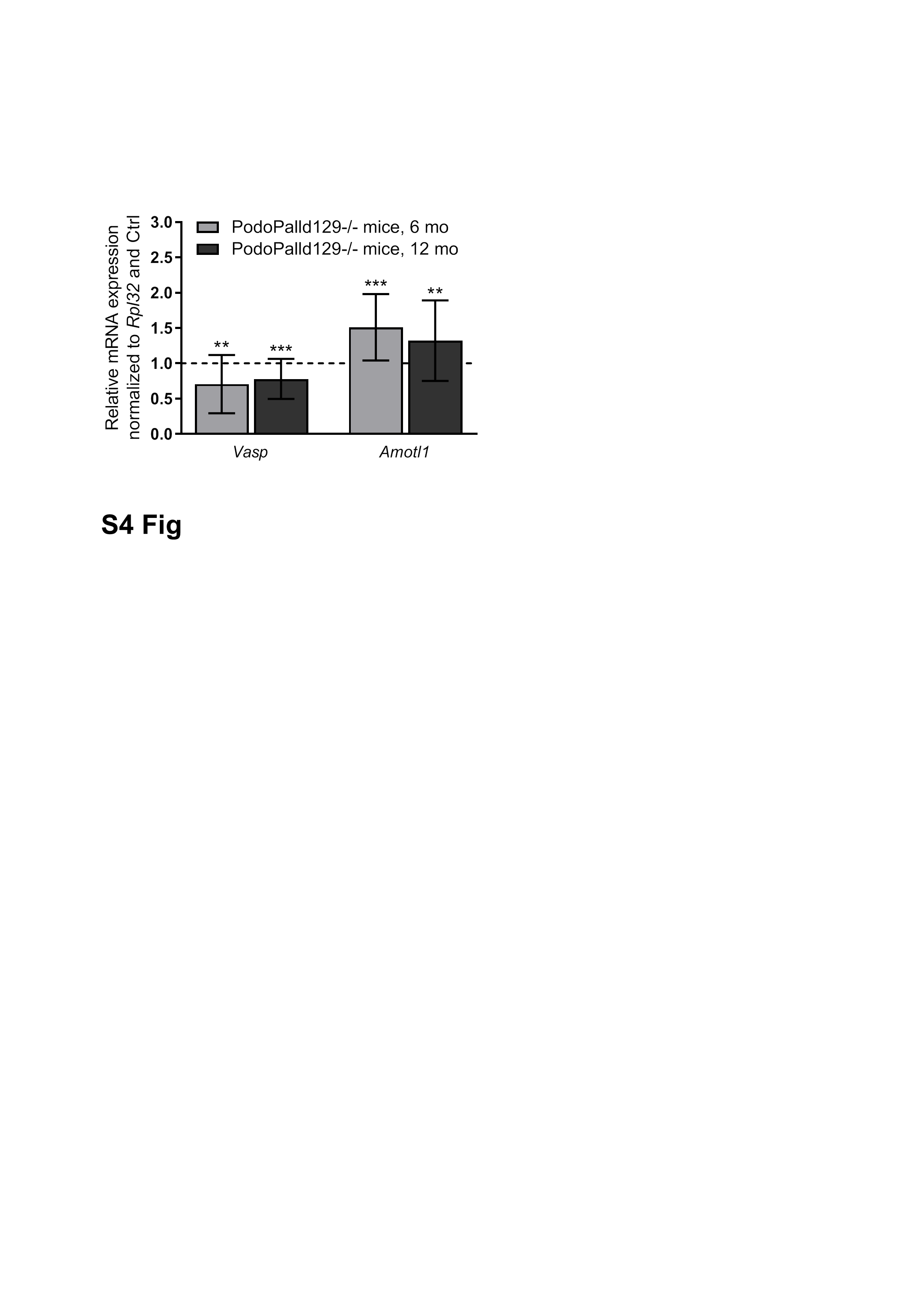

Supplement: S4 Fig — A significant downregulation of Vasp mRNA and upregulation of Amotl1 mRNA in PodoPalld129-/- glomeruli was found by qRT-PCR. Data are presented as means ± SD; ** p<0.01; *** p<0.001; unpaired Student’s t-test. (TIF) [file pone.0260878.s004.tif]

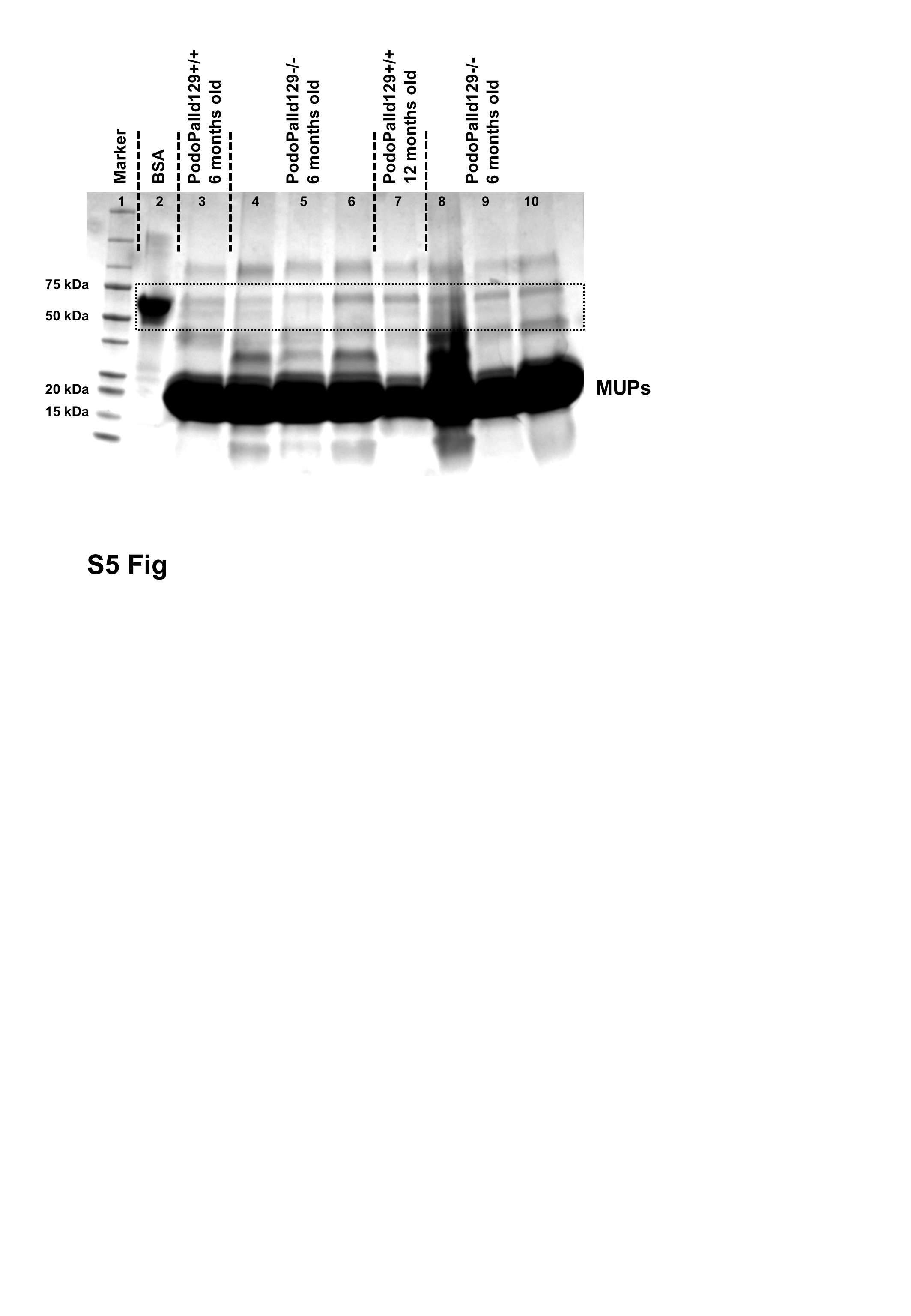

Supplement: S5 Fig — Equal volume of sterile urine from 6- and 12-months old PodoPalld129+/+ and PodoPalld129-/- mice were separated by SDS-Page and were subsequently stained using CBB (Coomassie Brilliant Blue). BSA was used as a charge control (lane 2). No albumin band is seen (dotted outline), in 6 months old PodoPalld129-/- (lane 4–6) as well as in 12 months old PodoPalld129-/- (lane 8–10) indicating no increased proteinuria in the PodoPalld129-/- mice. The major urinary proteins (MUPs) showed a strong signal between 15 kDa and 20 kDa. (TIF) [file pone.0260878.s005.tif]
