# Supplementary material for: The podocyte-specific knockout of palladin in mice with a 129 genetic background affects podocyte morphology and the expression of palladin interacting proteins
Source: PLoS One. 2021 Dec 8;16(12):e0260878. doi: 10.1371/journal.pone.0260878 (PMC8654177; doi:10.1371/journal.pone.0260878)
Supplement: S1 Table — (DOCX) [file pone.0260878.s006.docx]

**Supplementary Table**

**Supplementary Table 1. Primer for RT-PCR and qRT-PCR**

| **Gene** | **SE/AS** | **Primer sequence** | **PCR-product** |
| --- | --- | --- | --- |
| **isolated glomeruli** | | | |
| *Palld*  *Nphs1*  *Nphs2*  *Pdlim2*  *Lasp-1*  *Ezrin*  *VASP*  *Amotl1*  *Rpl32* | SE  AS  SE  AS  SE  AS  SE  AS  SE  AS  SE  AS  SE  AS  SE  AS  SE  AS | 5’-CTCTCGATCACGGGACAGTGGAGAT-3’  5’-GTATCCATCAGCAACCCCCGTGTTC-3’  5'-GCCACCACCTTCACACTGAC-3‘  5’-AGACCACCAACCGCAAAGAG-3‘  5'-TCCGTCTCCAGACCTTGGAA-3‘  5'-GGCTTTGGACACATGGGCTA-3‘  5'-CCAAGGCAGTCCAGCTCTTTT-3‘  5'-GTGTGGAGCTTGGGTGGAGT-3‘  5'-TTTCACTGCGAGACCTGCAA-3‘  5'-TGTTGCTTGAGGCGGAGATT-3‘  5'-TATGCCGTTCAGGCCAAGTT-3‘  5'-CTGGTCCCTGCTGAGCTTGT-3‘  5'-AGGTCTGGGGCCTCAACTTC-3‘  5'-CTCTGGGGAGGGACCATTCT-3‘  5'-TCAGCCTGCGAGAACAGAAG-3‘  5'-GAGAGGACATGGGGCTATGC-3‘  5'-AGTTCATCAGGCACCAGTCAG-3′  5'-ATCAGGATCTGGCCCTTGAAC-3′ | 403 bp  233 bp  140bp  149 bp  143 bp  120 bp  149 bp  125 bp  109 bp |
| **genotyping mice** | | | |
| Cre(23)  Pod-Cre | SE  AS  SE  AS | 5’-GCATTACCGGTCGATGCAACGAGTGAT-GAG-3’  5’-GAGTGAACGAACCTGGTCGAAATCAGTGCG-3’  5’-GGTTGGCACCCCTCTAGCATGACATTAGGA-3’  5’-TCATCACTCGTTGCATCGACCGGTAATGCA-3’ | 408 bp  ~500 bp |
| (kindly provided by Marcus J. Moeller, Germany) | | | |
| Palld_PLGP3  Palld_PLGP4 | SE  AS | 5’-GCTTCGCTTCAAGGAGGACCTTCTG-3’  5’-TGTATATCATGTTGTGGTGTCAGCC-3’ | ~500 bp |
